# Pressure Leaching of Copper Slag Flotation Tailings in Oxygenated Sulfuric Acid Media

**DOI:** 10.1021/acsomega.2c02903

**Published:** 2022-09-26

**Authors:** Abdullah Seyrankaya

**Affiliations:** Department of Mining Engineering, Mineral Processing Division, Dokuz Eylul University Engineering Faculty, Buca, Izmir 35390, TÜRKİYE

## Abstract

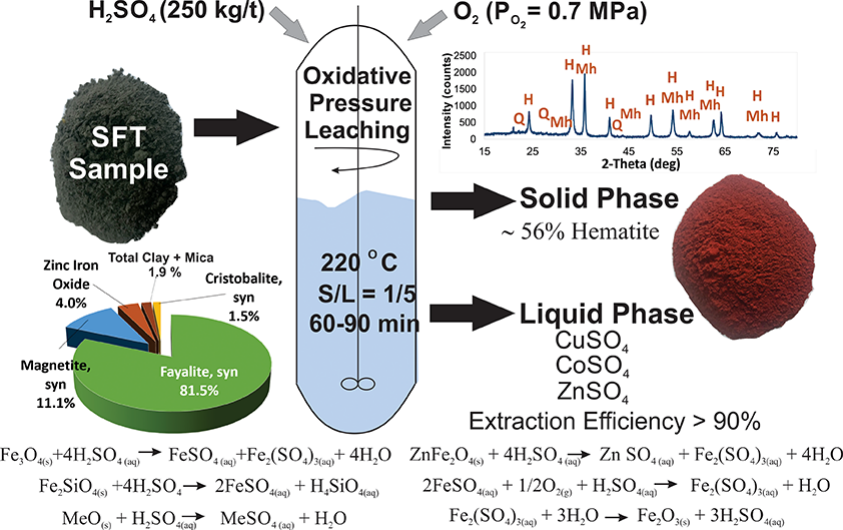

In this study, a hydrometallurgical method for the recovery of copper, cobalt, and zinc from copper slag flotation tailings (SFT) was investigated. SFT contains large amounts of valuable metallic compounds, such as copper, cobalt, and zinc. A representative SFT sample containing 0.50% Cu, 0.148% Co, 3.93% Zn, and 39.50% Fe was used in experimental studies. High-pressure oxidative acid leaching of SFT was carried out to assess the effects of sulfuric acid concentration, oxygen partial pressure, reaction time, solid/liquid ratio, and temperature on the extraction of copper, cobalt, zinc, and iron. The dissolution of metals from the SFT sample increased with temperature and sulfuric acid concentration. However, high acid concentrations and high solid/liquid (*S*/*L*) ratios led to gel formation that caused filtration problems and inhibited metal dissolution. The optimum leaching conditions were found to be a leaching time of 90 min, an acid concentration of 250 kg/t, a temperature of 220 °C, an *S*/*L* ratio of 1:5, and an oxygen partial pressure of 0.7 MPa. Under these conditions, 93.1 ± 1.1% Cu, 96.3 ± 1.8% Co, and 92.3 ± 1.7% Zn were extracted. Iron dissolution was only 0.5 ± 0.1%. This hydrometallurgical process almost completely recovers valuable metals. In particular, cobalt, which is of great importance in the production of lithium-ion batteries, has been declared a critical metal by the United States, Canada, and the EU and was taken into solution with very high extraction efficiency (>95%). Additionally, oxygen partial pressure enhanced copper, cobalt, and zinc dissolution. When O_2_ was not introduced into the leaching system, the extraction efficiencies of Co, Cu, and Zn were approximately 24.5, 5.3, and 26.3%, respectively, after 2 h of leaching treatment.

## 1 Introduction

Metals were discovered and first used approximately 10 000 years ago. Copper was the first metal used as a substitute for stone by humans and is still an important metal in industry today. Smelting is the pyrometallurgical process used to produce copper metal with the use of mining concentrates or copper scrap as the primary source of feed. In 2019, world copper production reached nearly 20.4 million tons.^1^ Afterward, it increased slightly to 21.0 million tons^1^ at the end of 2021 due to mines returning to full production, as well as the ramp-up of new mines starting in 2021. Copper slag is a solid byproduct obtained during the matte smelting, converting, and refining of copper. It has been estimated that for every ton of copper produced, approximately 2.2–2.5 tons of slag is generated as a result of the relatively low grades of copper concentrates now available.^2^ There are several copper smelting plants throughout the world, and this has resulted in the production of approximately 40 million tons per year of slag, which is regarded as waste.^3−5^ This slag is generally disposed of near smelter sites.^6,7^ Although the properties of copper slag in flash smelting, reverberatory furnace smelting, and other processes are generally similar, these slags can have different characteristics depending on how they are cooled from the smelter.^8^ When copper slag is crystalline, the major phases are usually fayalite (Fe_2_SiO_4_), along with other silicates. However, copper-containing phases in slag can differ, and they may be in the form of oxides, sulfides,^6^ or a mixture of both. One of the other main components of slag is the silica phase, which consists of both fayalite and glassy silicate phases. Other metals in slag, such as Ni, Co, and Zn, generally bond to silicon or iron to form silicate and ferrite phases instead of forming independent mineral compounds in the slag.^9,10^ Typical smelting slag contains approximately 30–45% FeO, 30–40% SiO_2_, 5–10% Al_2_O_3_, 2–6% CaO, and 2–4% MgO.^11^ Slags can contain significant quantities of valuable metals, such as cobalt, nickel, zinc, and copper, in various forms. In the last few decades, there has been growing interest in hydrometallurgical processes to recover valuable metals from copper smelting slags due to selective metal recovery, low energy consumption, low cost, less emission of toxic gas, and possible recovery of leachants. Hydrometallurgical processes which include leaching (acid leaching, alkaline leaching, oxidative leaching, water leaching, pressure leaching, and bioleaching), ion exchange, chelating, adsorption, precipitation, and solvent extraction are successfully applied to recover precious metals from various wastes.^12−22^ Furthermore, Lin and Chiu^23^ showed that hydrometallurgy offers a possibility and an opportunity to convert used dry batteries into pure metals or metal salts with little energy needed. In particular, cobalt is a metal that is absolutely critical in battery storage for electric vehicles. It is relevant that cobalt-bearing tailings are of particular importance because cobalt has been deemed a “critical metal” by the United States, Canada, and the European Union (EU) based on its relatively high economic importance and supply risk. Lithium-based batteries, such as LCO (LiCoO_2_), LMO (LiMn_2_O_4_), LTO (Li_2_TiO_3_), NCA (LiNiCoAlO_2_), NMC (LiNiMnCoO_2_), and LPF (LiFePO_4_) batteries, which use various combinations of anode and cathode materials, are currently the most widely used batteries in electric vehicles.^24^ NCA and NMC batteries in particular have very high market shares in electric vehicles. For example, the average 100 kWh lithium-ion battery pack (NCA) used to power a Tesla Model X has approximately 20 kg of cobalt. For this reason, cobalt has become an essential metal in the rechargeable battery manufacturing and electric car industries.

Several researchers have investigated the extraction of metals from slags using various extractants, such as ferric chloride,^25,26^ ferric sulfate,^27,28^ ammonium chloride,^29^ chlorine solution,^30,31^ sulfuric acid,^27,32−38^ hydrochloric acid,^33,39^ ammonium hydroxide,^33^ nitric acid,^40,41^ and aqua regia,^42−47^ as leaching agents. Sulfating or chloride roasting can be applied to convert sulfide phases to soluble sulfate compounds prior to water or dilute acid leaching. Ammonium chloride was investigated as a chloride agent,^48^ whereas sulfating agents included ferric sulfate,^49^ ammonium sulfate,^50^ sodium sulfate,^51^ and sulfuric acid.^10,52−55^ To improve extraction efficiency, additional treatments in leaching systems, such as adding oxidants (H_2_O_2_, K_2_Cr_2_O_7_, and NaClO_3_),^33−35,39,56−59^ high-temperature leaching^25,27,28,33,34,38,48,53^ or applying oxidative pressure,^32,37,60,61^ have also been investigated. Moreover, Potysz et al.^62^ and Tian et al.^63^ presented detailed reviews on the recovery, leaching, and environmental evaluation of precious metals from copper slags, as well as the formation mechanism of slags and their chemical, physical, and phase composition.

When the copper in the slag is largely in sulfide phases, flotation of copper slag is similar to sulfide ore flotation of copper.^64−69^ In the flotation of copper slag, oxides, alloys, and metallic phases containing Co and Ni are generally depressed and concentrated in the tailings.^6^ Hence, copper slag flotation tailings (SFT) may contain copper as well as substantial amounts of cobalt and other metals.^58^ Some researchers have studied the extraction of metals from SFT by sulfuric acid leaching in the presence of oxidants.^28,58,70,71^

In this study, the extraction of Cu, Co, and Zn from SFT by hydrometallurgical treatment based on high-pressure oxidative leaching in sulfuric acid media was investigated. The presence of cobalt may add to the economic value of SFT. The oxidant is one of the most important factors for the decomposition of fayalite and magnetite in the leaching system. Parameters affecting the recovery efficiency, such as the leaching temperature, sulfuric acid concentration, oxygen partial pressure, leaching time, and solid/liquid (*S*/*L*) ratio, were investigated. In addition to the extraction efficiencies of copper, cobalt, and zinc, the leaching behavior of iron was examined under autoclave conditions.

## 2 Experimental Section

### 2.1 Materials

**Figure 1:**
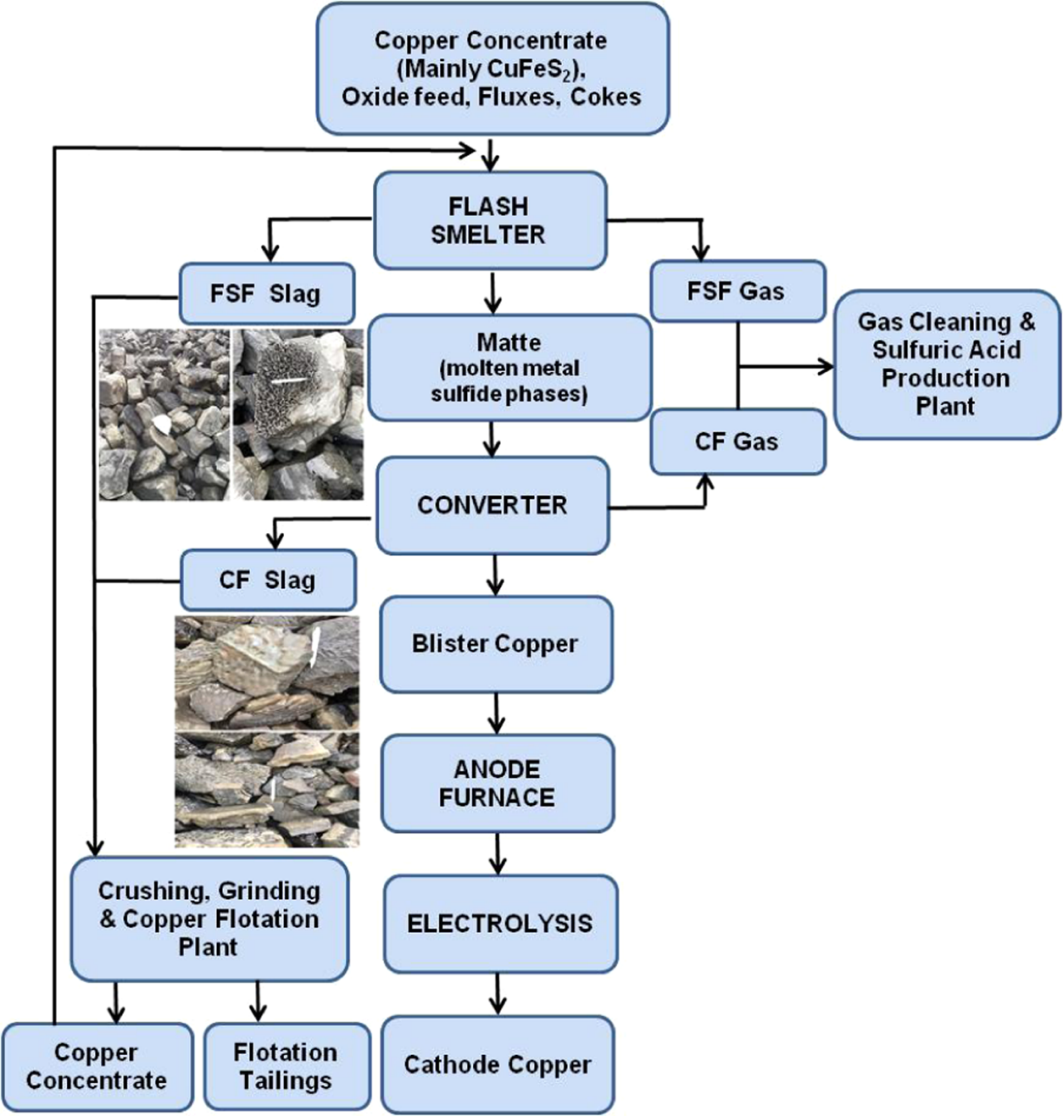
Simplified flowchart for the copper smelting process in the Eti Bakır Company (FSF: flash smelting furnace, CF: converter furnace).

The experimental work was carried out on a representative sample of SFT obtained by the mining company Eti Bakır (Türkiye). The Eti Bakır Company basically consists of six main facilities (Figure 1): a copper smelter plant, a flotation plant, a sulfuric acid production plant, an electrolysis plant, an oxygen plant, and a crystallized ammonium sulfate plant. In the copper smelter plant, 99.9% pure cathode copper is produced using flash furnace technology. A simplified flowchart of the plant is given below. In the plant, flash and converter furnace slags are mixed in certain proportions and then subjected to a flotation process. The concentrate containing copper is sent back to the flash furnace. The SFT contains considerable amounts of copper, cobalt, and zinc.

**Figure 2:**
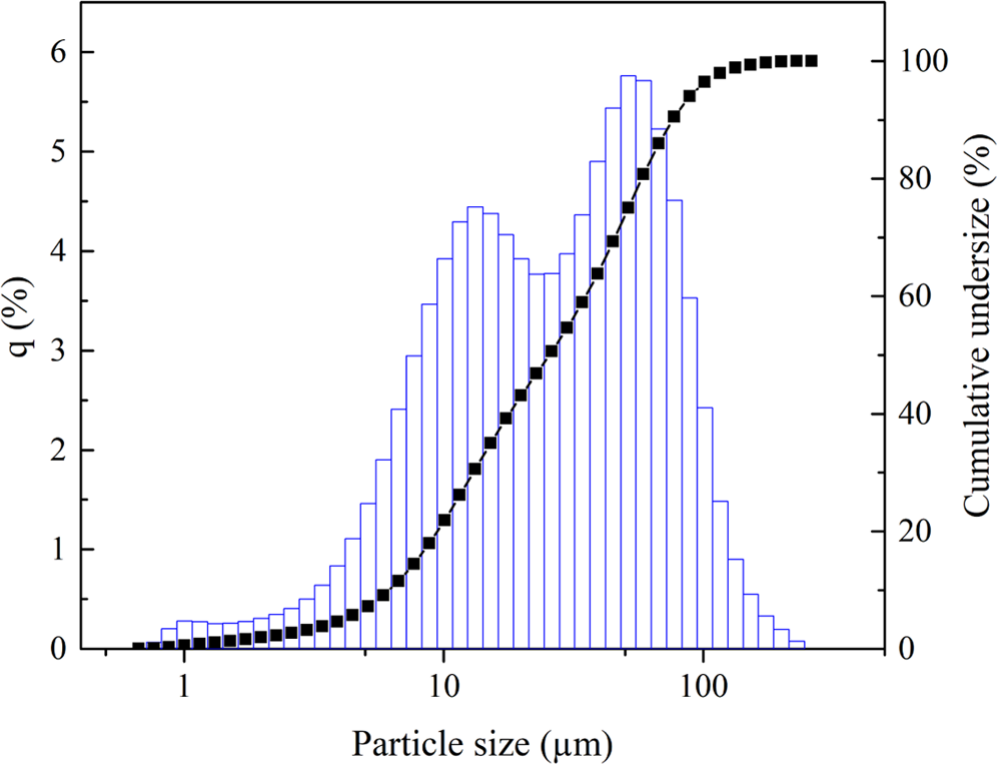
Particle size distribution plot of the slag sample. The *y*-axis *q*(%) indicates the amount of each size by volume.

The SFT sample and leach residue were characterized by X-ray diffraction (XRD). The XRD patterns were recorded on a Rigaku X-ray diffractometer using Cu Kα radiation with a scanning rate of 2° min^–1^ from 3 to 80°. The generator voltage and current were 40 kV and 30 mA, respectively. Rietveld refinement analysis using X’Pert HighScore Plus software (PANanalytical) was performed to obtain the percentages of different phases in the samples. X-ray photoelectron spectroscopy (XPS) analyses were performed with a Thermo Scientific K-Alpha using an Al Kα X-ray source (microfocused monochromator) high-performance XPS spectrometer. Survey scans for the detection of all elements were carried out at a pass energy of 150 eV and a step size of 1 eV. The electron energy analyzer was operated with a pass energy of 30 eV and a step size of 0.1 eV, enabling high-resolution spectra to be obtained. Grain size analysis was performed using a Partica LA-950V2 laser diffraction particle size distribution analyzer (Horiba) in wet mode. According to the particle size distribution curve (Figure 2), *d*_80_ and the mean particle size of the SFT sample were determined to be 58 and 35 μm, respectively. Elemental analysis of the filtrate or solid sample was performed by inductively coupled plasma optical emission spectrometry (ICP–OES) (Varian 710-ES). All chemical reagents (Merck) used in the pressure leaching experiments were of analytical grade. The samples used in the leach tests contained averages of 0.50% Cu, 0.15% Co, 3.93% Zn, 1.53% Al, 0.57% Ca, 0.14% S, and 39.50% Fe. The full chemical analysis results of the SFT sample are given in Table 1.

**Table 1:** Chemical Analysis Results for the SFT Sample

element	unit	SFT sample content	element	unit	SFT sample content	element	unit	SFT sample content
Ag	ppm	2	K	%	0.70	Sc	ppm	<10
Al	%	1.53	La	ppm	<50	Sr	ppm	120
Ba	ppm	2340	Mg	%	0.35	Th	ppm	<50
Bi	ppm	40	Mn	ppm	230	Ti	%	0.07
Ca	%	0.57	Mo	ppm	480	Tl	ppm	50
Cd	ppm	50	Na	%	0.23	U	ppm	<50
Co	ppm	1480	Ni	ppm	10	V	ppm	60
Cr	ppm	480	P	ppm	90	W	ppm	<50
Cu	ppm	5000	Pb	ppm	3230	Zn	%	3.93
Fe	%	39.5	S	%	0.14	SiO_2_	%	29.27
Ga	ppm	<50	Sb	ppm	240			

**Figure 3:**
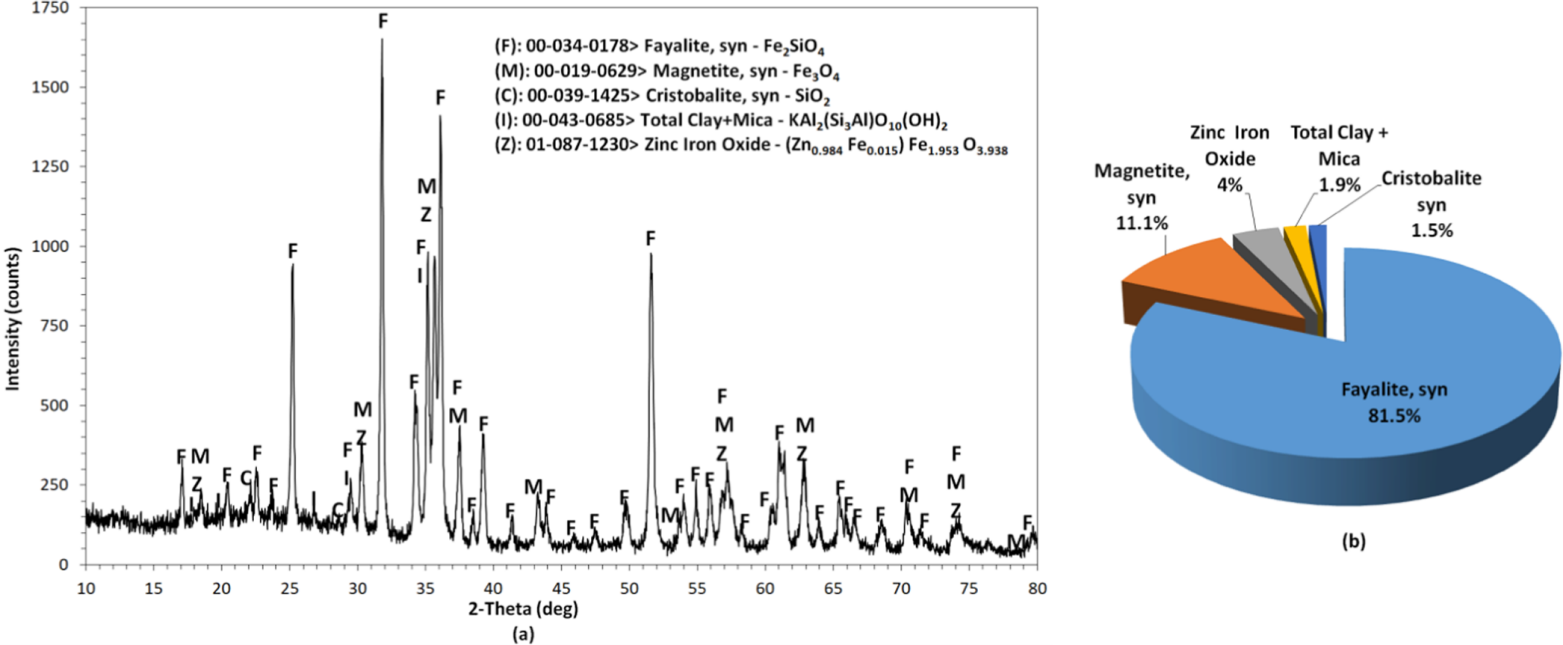
X-ray diffraction pattern (a) and distribution of phases (b) of the SFT sample.

The elemental composition and chemical oxidation states of surface and near-surface species can be detected by XPS analysis. Therefore, XPS analysis was conducted to assess the chemical states of both the SFT sample and leaching residue. Many clear peaks summarized in Table 2 were observed for the SFT sample. No sulfur (S) peak was detected by XPS because of the high flotation recovery of sulfide minerals in the copper slag prior to leaching (Figure 1). For this reason, copper, cobalt, and zinc in the flotation tailings were mostly in the oxide-silicate or metallic forms. According to mineralogical examination, the SFT sample contained mainly fayalite (Fe_2_SiO_4_, 81.5%), magnetite (Fe_3_O_4_, 11.1%), zinc iron oxide (franklinite, (Zn_0.984_Fe_0.015_)Fe_1.953_O_3.938_, 4.0%), cristobalite (SiO_2_, 1.5%), and clay-mica (KAl_2_(Si_3_Al)O_10_(OH)_2_, 1.8%) (Figure 3).

**Table 2:** X-ray Photoelectron Spectroscopy (XPS) Analysis Result of the SFT Sample

peak name	binding energy (eV)	FWHM (eV)	area (P) CPS.eV	atomic (%)
O 1s	531.96	3.21	738 994.9	55.13
Zn 2p	1022.25	2.77	195 546.2	1.6
Fe 2p	712.16	5.74	398 700.9	2.34
Si 2p	102.96	2.94	87 565.9	16.81
Mg 1s	1304.14	2.87	40 407.6	2.02
C 1s	285.12	2.79	67 348.4	14.42
Cu 2p	935.01	3.97	154 657.3	0.67
Pb 4f	139.20	2.94	51 013.4	0.19
Cl 2p	199.44	1.82	6634.6	0.45
K 2p	294.39	1.65	10 193.0	0.5
Co 2p	784.36	10.12	90 426.8	0.41
Al 2p	75.03	4.28	9764.5	4.61
Ca 2p	351.77	3.10	14 037.0	0.42
Na 1s	1072.20	1.59	5290.3	0.43

### 2.2 Method

**Figure 4:**
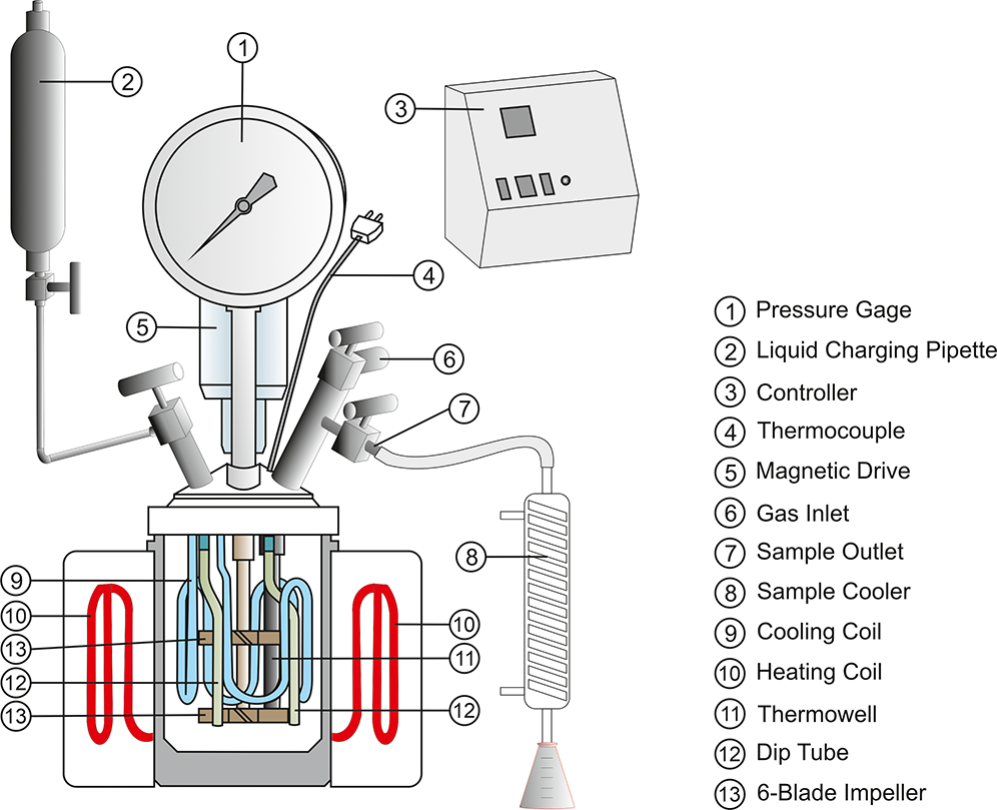
Experimental setup for pressure leaching.

The pressure leaching experiments were conducted in a 1 L titanium autoclave (Parr, Inc.,). A schematic diagram of the autoclave system with a heating mantle, PID temperature controller, variable speed stirrer, sampling dip tube, and internally mounted serpentine-type cooling coil is given in Figure 4.

The experiments were carried out in batch mode using 100, 150, 200, and 250 g of SFT (*d*_80_ = 58 μm) and various concentrations of sulfuric acid, at oxygen pressure (*P*_O_2__) of 0.7 MPa. The reaction vessel was first preheated for approximately 60–70 min. Then, oxygen and acid were added at the preset temperature, and the oxygen partial pressure was adjusted to the desired level and maintained constant for the duration of the experiment. The stirring rate was kept constant at 500 rpm during the test. In the experiments, 10–15 mL of slurry was sampled by a sampling dip tube. The slurry was cooled immediately, centrifuged, and filtered with a 0.45 μm PTFE syringe filter. After 2 h of residence time, the oxygen flow was shut down, and the autoclave was water-cooled to less than 60 °C. After solid–liquid separation by vacuum filtration, the solid was washed with deionized water several times. The leaching residues were dried for at least one day at 80 °C. Elemental analysis of the filtrate or solid residue was performed by ICP.

The percentage extraction efficiency of cobalt, copper, zinc, and iron during leaching was calculated according to the following formula
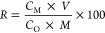
1where *R* (%) is the extraction efficiency of metal (Co, Cu, Zn, or Fe); *C*_M_ (g/L) is the elemental concentration determined by ICP–OES in the leachate samples; *V* (L) is the total volume of the acid leaching solution; *C*_O_ (%) is the metal content of Co, Cu, Zn, or Fe in the slag sample; and *M* (g) is the mass of slag used.

Moreover, six additional tests were conducted under optimal leaching conditions for repeatability, and the percent extraction of metals was reported as the average ± standard deviation.

## 3 Results and Discussion

### 3.1 Effect of H_2_SO_4_ Concentration

**Figure 5:**
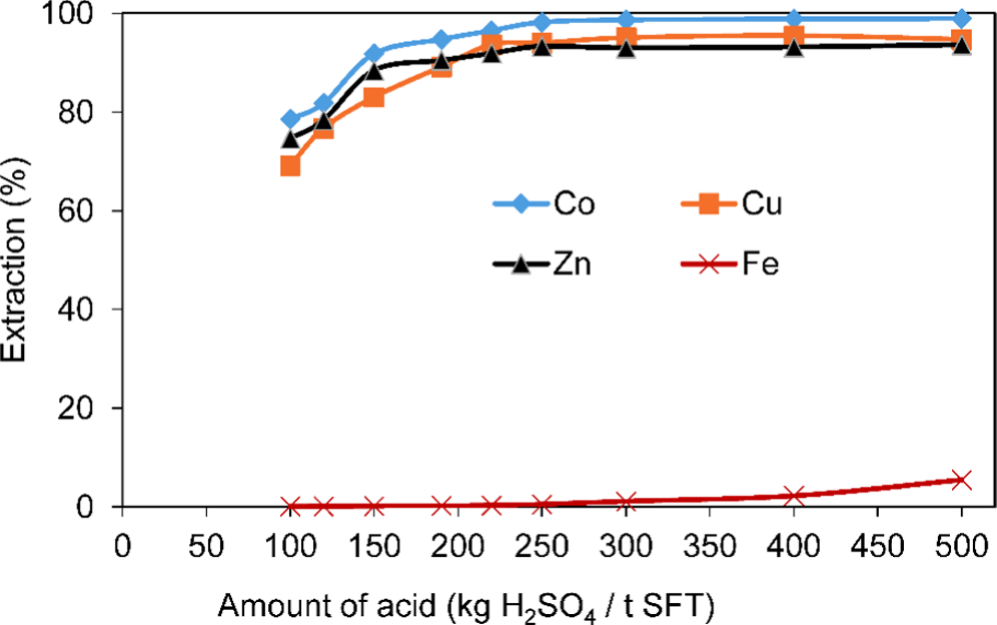
Effect of sulfuric acid addition amount on metal extraction efficiency; *S*/*L* = 1:5, *t* = 220 °C, *P*_O_2__ = 0.7 MPa, τ = 120 min, *d*_80_ = 58 μm.

A series of high-pressure leaching experiments were carried out by varying the addition amount of sulfuric acid from 100 to 500 kg/t SFT at 220 °C with a leaching time of 2 h, an *S*/*L* ratio of 1:5 (i.e., 1 kg SFT sample and 5 L liquid). The results are shown in Figure 5. The dissolution of metal increased significantly with increasing sulfuric acid concentration. The extraction efficiency of Cu, Co, and Zn improved with increasing sulfuric acid concentration up to 250 kg/t. Figure 5 shows that the extraction of cobalt, copper, and zinc increased from 78.6 to 98.2%, 69.2 to 94.7%, and 74.7 to 93.3%, respectively, when the initial acid concentration increased from 100 to 250 kg/t (corresponding to 20 and 50 g/L). The effect of adding more acid on the leaching efficiency of base metals was limited. However, when the acid concentration was higher than 250 kg/t, iron dissolution significantly increased (from 0.1 to 5.5%) with increasing initial acid concentration because of the redissolution of hematite formed in the leaching residue, which increases as the amount of acid added increases. There is a positive correlation between extraction efficiency and acid concentration, meaning that stronger acidity enhances metal extraction.^32,33,35,37,38,53,59^ Consequently, further tests were carried out with the addition amount of sulfuric acid fixed at 250 kg/t to achieve the highly selective leaching of Co, Cu, and Zn to inhibit the Fe dissolution and entry into the leaching solution. Moreover, experimental studies of metal extraction with strong acids also showed an important limitation due to the formation of silica gel (eq 2), which makes metal extraction and pulp filtration much more difficult.^32,34,53,56,72^
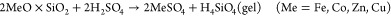
2

### 3.2 Effect of Leaching Temperature on Metal Extraction

**Figure 6:**
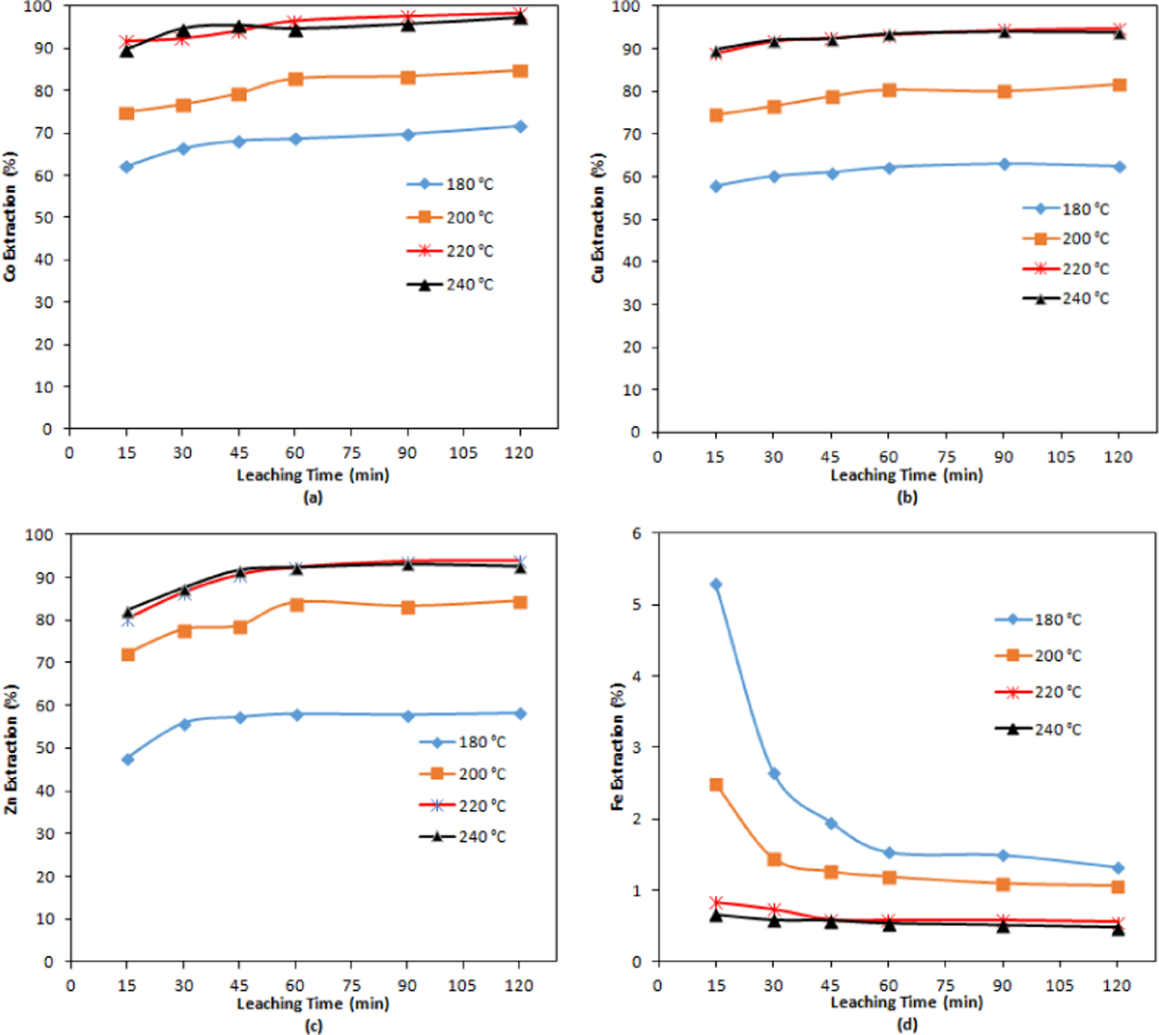
Effect of temperature on metal extraction: (a) cobalt, (b) copper, (c) zinc, and (d) iron, H_2_SO_4_ = 250 kg/t, *S*/*L* = 1:5, *P*_O_2__ = 1.2 MPa, τ = 120 min, *d*_80_ = 58 μm.

The leaching temperature also plays a significant role in metal extraction. Figure 6 shows the effect of leaching temperature on metal extraction with an acid addition amount of 250 kg/t, leaching time of 120 min, and *S*/*L* ratio of 1:5. Figure 6 shows that the extraction efficiency of cobalt, copper, and zinc was significantly affected by changes in temperature from 180 to 240 °C and that the maximum metal extraction was obtained at 220 °C. Further increasing the temperature had a slight influence. The dissolution temperature was found to be the most effective factor controlling the dissolution kinetics during oxidative pressure acid leaching. Increasing the temperature had an increasing effect on cobalt, copper, and zinc leaching recovery. At 220 °C, the extraction efficiencies of cobalt, copper, and zinc reached 96.4, 93.3, and 92.2% in the first 60 min, respectively. Similar findings were reported by Liao et al.^73^ They stated that when the temperature increased from 140 to 200 °C, under a H_2_SO_4_ concentration of 0.4 mol/L, *S*/*L* ratio of 1:6, and 0.6 MPa, the leaching efficiency of Cu increased from 58.3 to 95.1% for the leaching of copper smelting slag. As shown in Figure 6d, the total iron extraction was 1.3% at 180 °C, 1.1% at 200 °C, 0.6% at 220 °C, and 0.5% at 240 °C after 2 h. Changing the leaching temperature under oxidative conditions and a certain *S*/*L* ratio had no significant effect on iron dissolution. In all cases, iron dissolution was less than 1.5% in 2 h. Moreover, iron in the fayalite, magnetite, and franklinite phases is easily dissolved into solution under acidic conditions. The oxidation of Fe^2+^ with oxygen gas is an integral part of the precipitation process. The hydrolysis of ferric iron is favored at high temperatures and low pHs (*P*_O_2__ > 0.5 MPa, *t* > 185 °C). Under this condition, while iron precipitation takes place via simultaneous oxidation of Fe^2+^ and hydrolysis of Fe^3+^, other ions remain in solution. Thus, hydrolysis is a very efficient way to selectively remove iron from solution. The reactions for the oxidation and hydrolysis of iron (hematite precipitation) in sulfate media are given by eqs 3–5.

Ferrous sulfate oxidation to ferric sulfate
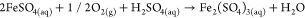
3

Ferric sulfate hydrolysis to hematite
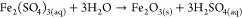
4
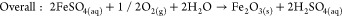
5

### 3.3 Effect of Oxygen Partial Pressure

**Figure 7:**
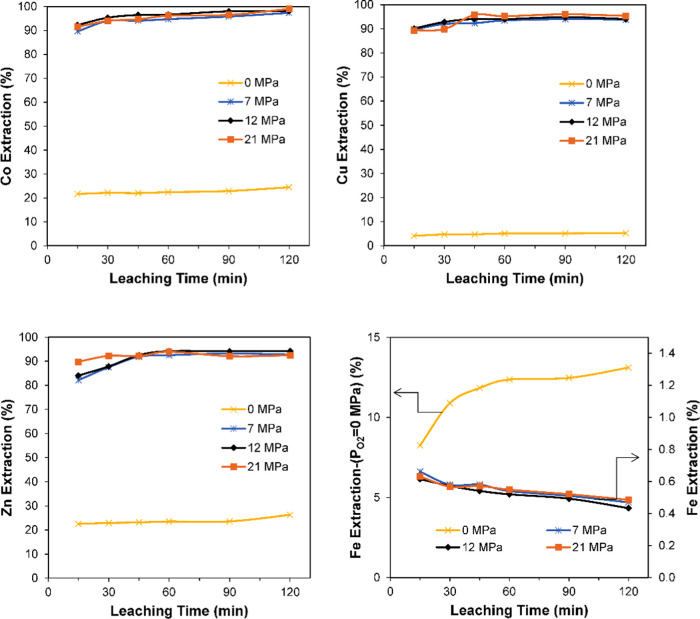
Effect of oxygen partial pressure on leaching of SFT; H_2_SO_4_ = 250 kg/t, *t* = 240 °C, *S*/*L* = 1:5, τ = 120 min, *d*_80_ = 58 μm.

Oxygen is the main oxidant in the high-pressure leaching process of slags and plays a decisive role in the leaching processes. Oxygen considerably affects not only the dissolution of some metals or metal minerals but also the oxidation and hydrolysis of iron in slag. The oxidation reaction of ferrous sulfate with oxygen gas occurs in two physicochemical steps: (a) the mass transfer of oxygen from gas into the liquid phase and (b) the homogeneous oxidation of ferrous sulfate with oxygen (see eq 3). The solubility of oxygen in water decreases gradually as the temperature rises (from 0 to 100 °C). However, the solubility of oxygen in water increases with increasing temperature above the boiling point of water. In addition, an increase in oxygen partial pressure causes a significant increase in oxygen solubility. The effect of oxygen partial pressure on the degree of leaching of the SFT sample was studied at a leaching temperature of 240 °C, an acid concentration of 250 kg/t, an *S*/*L* ratio of 1:5, a particle size of 58 μm, and time of 120 min. Figure 7 shows the variations in the extraction efficiencies of cobalt, copper, and zinc as a function of oxygen partial pressure in the range of 0–2.1 MPa. Figure 7 shows that with increasing oxygen partial pressure, the dissolution of copper, cobalt, and zinc increased. At a 0.7 MPa oxygen partial pressure, the extraction efficiencies of cobalt, copper, zinc, and iron were 97.4, 93.9, 92.7, and 0.5%, respectively, whereas extraction efficiencies of 24.5, 5.3, 26.3, and 13.1% were achieved in the experiment without oxygen supply. It can be concluded from the results presented in Figure 7 that the optimal partial pressure of oxygen is 0.7 MPa and a further increase did not significantly change the degree of leaching of any of the metals. In addition, oxygenated conditions appear to be a factor promoting metal extraction with the simultaneous accomplishment of a low iron extraction efficiency.^32−34,37,56^ Increased oxygen pressure greatly improves the dissolved oxygen content in solution and increases the gas–liquid contact area, thereby accelerating the oxidation rate of Fe^2+^ to Fe^3+^, realizing rapid iron precipitation of the leaching solution and enhancing base metal extraction. Moreover, an increase in oxygen pressure accelerates the oxidation reactions of sulfide forms such as CuS, Cu_2_S, Cu_9_S_5_, Cu_5_FeS_4_, and CuFeS_2_ that can exist in copper slag or SFT. Altundogan et al.^35^ used potassium chromate (K_2_Cr_2_O_7_) as an oxidant in sulfuric acid leaching of converter copper slag. They concluded that oxidant addition improves copper leaching, whereas it has adverse effects on the extraction of Co, Zn, and Fe. Urosevic et al.^71^ studied the effect of ferric sulfate or hydrogen peroxide on the leaching of copper slag and SFT using sulfuric acid. They reported that the highest copper extraction efficiency (63.4% when using 3 M H_2_O_2_ and 1 M H_2_SO_4_) was attained with hydrogen peroxide at room temperature. Banza et al.^34^ investigated hydrogen peroxide as an oxidant in sulfuric acid media. According to the results reported in this work, H_2_O_2_ addition to the leaching system considerably decreased iron dissolution from 90% to less than 5%, while it increased copper recovery from 60 to 85% at 80 °C and did not affect cobalt or zinc recovery. The effect of hydrogen peroxide on the extraction of metals in sulfuric acid solutions using copper smelter flotation tailings was also studied by Yiğit et al.^58^ They reported that a high leaching efficiency was achieved for copper (100%), zinc (86.3%), and iron (94.6%), but the extraction of cobalt was consistently limited to ≤10.7% even with a fine size (*d*_80_ = 27 μm). High-pressure oxidative acid leaching of copper converter slag,^32^ converter slag, and pyrrhotite tailings,^60^ nickel smelter slags,^74^ and historical copper slag^75^ yielded high leaching efficiencies in the range of 91–99% for valuable metals such as Ni, Cu, Co, and Zn. Recently, a study on the kinetics of copper extraction from copper smelting slag by pressure oxidative leaching in sulfuric acid solution was presented by Shi et al.^76^ They reported that different leaching stages have different controlling steps according to the shrinking core model: leaching is controlled by chemical reactions in the early stage, then mixed control occurs, and finally leaching is controlled by diffusion of the solid product layer. They found that the apparent activation energies of the chemical reaction-controlled and solid product layer diffusion-controlled processes were 47.3 and 11.35 kJ/mol, respectively.^76^ In the present work, a high extraction efficiency (>92%) and selective dissolution of base metals for Co, Cu, and Zn were achieved within 45–60 min at 220 °C and a 50 g/L initial H_2_SO_4_ concentration. The general reactions for the leaching of Cu, Co, Ni, and Zn in slag can be written as follows^60,61,75^

Metal/metal oxide/sulfide/silicate (Me = Cu, Co, Zn, Ni, Fe) leaching by acid
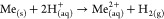
6
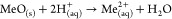
7
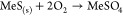
8
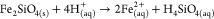
9
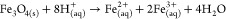
10
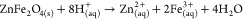
11

Fayalite, magnetite, and franklinite are dissolved by sulfuric acid, releasing ferrous and ferric iron into solution (eqs 9–11).

### 3.4 Effect of Solid/Liquid (*S*/*L*) Ratio on Metal Extraction

**Figure 8:**
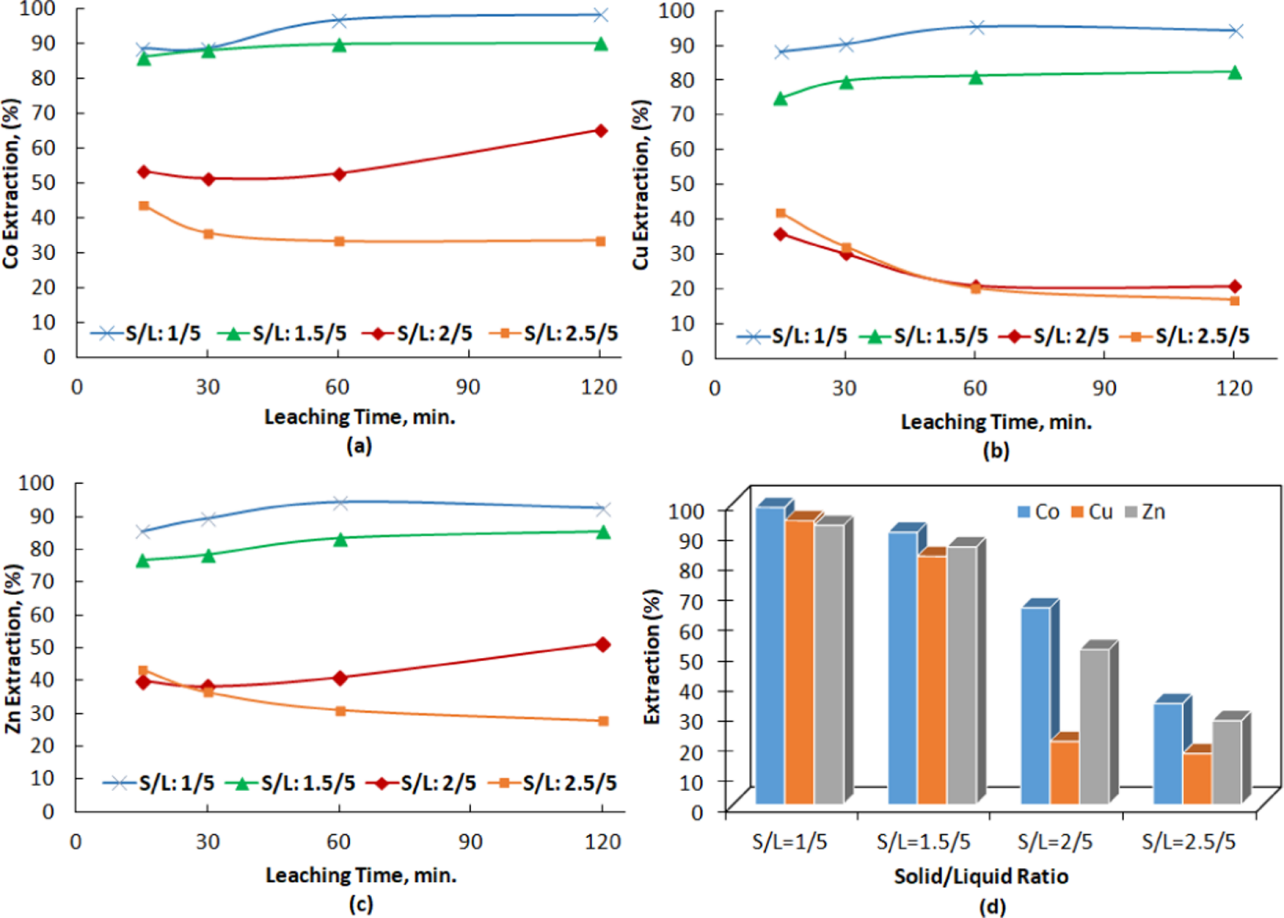
Effect of solid/liquid ratio (w/v) on the extraction kinetics of cobalt (a), copper (b), and zinc (c). Extraction percentages at different solid/liquid ratios. At the end of leaching time (2 h) (d), H_2_SO_4_ = 250 kg/t, *P*_O_2__ = 0.7 MPa, *t =* 220 °C, and *d*_80_ = 58 μm.

**Figure 9:**
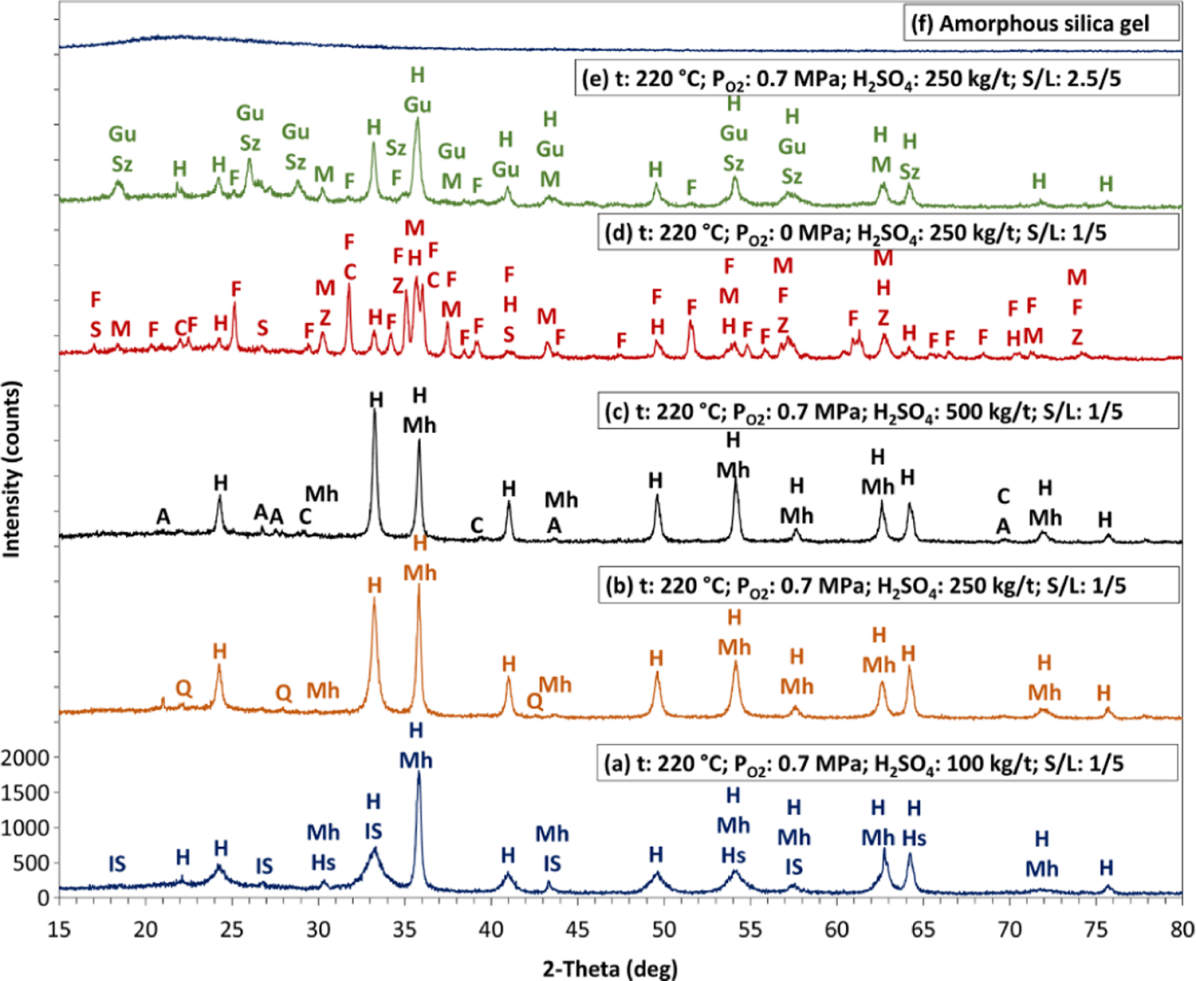
XRD patterns of selected pressure leaching residues (H: α-hematite, Hs: hercynite, Mh: maghemite (γ-hematite), IS: iron silicate, Q: quartz, C: coesite, A: anglesite, F: fayalite, M: magnetite, C: cristobalite, Z: Franklinite (zinc iron oxide), S: sillimanite, Gu: gunningite, Sz: szomolnikite, I: illite).

The *S*/*L* ratio used in metal extraction is one of the most important parameters for designing process equipment. Its optimum value depends on other parameters as well. Usually, higher recovery efficiencies can be achieved when the pulp density is lower due to the greater contact of the leachate with the surface of solid particles. The effect of *S*/*L* ratio on the dissolution of SFT was investigated under different *S*/*L* ratios (1:5, 1.5:5, 2:5, and 2.5:5). To obtain the desired ratio, the liquid volume was kept constant, and the amount of slag was changed. Figure 8a–c presents the extraction results for cobalt, copper, and zinc with respect to leaching time while Figure 8d shows metal extraction versus *S*/*L* ratio. Figure 8 shows that the extraction of Co, Cu, and Zn increased with a decrease in the amount of solids. The maximum extractions for Co, Cu, and Zn (>90%) were obtained at an *S*/*L* ratio of 1:5. The extraction efficiencies of Co, Cu, and Zn decreased sharply when the *S*/*L* ratio increased from 1.5:5 to 2:5 or 2.5:5. As the *S*/*L* ratio increases, the slurry density gradually increases, decelerating mass transfer, and therefore negatively affects slag dissolution. The cobalt, copper, and zinc leaching efficiencies decreased from 98.6, 94.2, and 92.6% to 33.5, 16.9, and 27.9%, respectively, as the *S*/*L* ratio increased from 1:5 to 2.5:5 at an acid concentration of 250 kg/t over 2 h. Moreover, the iron concentration in solution increased from 0.5 to 12.5 g/L with an increase in *S*/*L* ratio from 1:5 to 2.5:5 by weight of solids, indicating incomplete oxyhydrolysis. When the amount of sulfuric acid was kept constant at 250 kg/t and the *S*/*L* ratio increased, the acid concentration in the solution changed. Increasing free hydrogen ions in solution with the increase in H_2_SO_4_ promoted more silica gel formation at high *S*/*L* ratios (*S*/*L* = 2:5 and 2.5:5) (eq 2). The generation of silica gel significantly inhibited metal extraction. At the end of the experiments with relatively high *S*/*L* ratios (2:5 and 2.5:5), all of the leached material was in a gelatinous form and had very little fluidity. The considerable decrease in the extraction of Co, Cu, and Zn at an *S*/*L* ratio of 2.5:5 might be due to the combined effect of higher slurry viscosity, less dissolved oxygen, and the formation of a larger quantity of gelatinous material, thus coating the particles. Similar behavior was also noted in the oxidative pressure leaching of a copper slag.^75^ Further confirmation was provided by XRD analysis of the leaching residue obtained with an *S*/*L* ratio of 2.5:5; the diffraction pattern is provided in Figure 9e. This pattern shows that the residue contained ZnSO_4_H_2_O, FeSO_4_H_2_O, fayalite, magnetite, and hematite.

### 3.5 Characterization of Leaching Residues

Extraction efficiencies of 96.3 ± 1.8% for cobalt, 93.1 ± 1.1% for copper, 92.3 ± 1.7% for zinc, and 0.5 ± 0.1% for iron were achieved under the optimum leaching conditions (H_2_SO_4_ = 250 kg/t, *S*/*L* = 1:5, *P*_O_2__ = 0.7 MPa, τ = 60 min, stirring rate of 500 rpm). In contrast to the results reported in previous studies, the optimized conditions caused high selective leaching of cobalt, copper, and zinc compared to iron, strongly indicating the thorough removal of iron from the leaching liquor.

The XRD patterns of the selected leaching residues are given in Figure 9, and the phase names and their formulas are summarized in Table 3. The leaching residue at a low acid concentration (100 kg/t, corresponding to 30 g/L) mainly included hematite (α-F_2_O_3_), but it also contained some undissolved fayalite (iron silicate, Fe_2_(Fe_0.565_Si_0.435_)O_4_) and maghemite (γ-Fe_2_O_3_) (Figure 9a) and a small amount of hercynite (Fe_0.882_Al_0.118_)(Al_1.882_Fe_0.118_)O_4_. The leaching residue obtained under the optimal leaching conditions contained very low amounts of base metal and 39.17% iron, and the main phase was α-Fe_2_O_3_ with a small amount of γ-Fe_2_O_3_ (maghemite) (approximately 56 wt % Fe_2_O_3_) (Figure 9b). At a high acid concentration (500 kg/t, corresponding to 100 g/L) and an *S*/*L* ratio of 1:5, high metal extraction was obtained, but iron dissolution was relatively high (5.5%). Under these leaching conditions, the leaching residue mainly contained hematite and small amounts of coesite (SiO_2_) and anglesite (PbSO_4_) (Figure 9c). Fayalite was not completely dissolved in the experiments performed in an oxygen-free environment, and the XRD analysis revealed that the leaching residue contained mainly fayalite, magnetite, zinc iron oxide (Zn_0.945_Fe_1.78_O_3.71_), and a small amount of hematite and sillimanite (Al_2_SiO_5_) (Figure 9d).

**Table 3:** Phases Detected in the XRD Patterns

leaching residue	name	formula	PDF number
(a)	hematite (H)	α-Fe_2_O_3_	01-089-8103
	hercynite (Hs)	(Fe_0.882_Al_0.118_)(Al_1.882_Fe_0.118_)O_4_	01-082-0585
	maghemite (Mh)	γ-Fe_2_O_3_	01-089-3850
	iron silicate (IS)	Fe_2_(Fe_0.565_Si_0.435_)O_4_	01-089-0842
(b)	hematite (H)	α-Fe_2_O_3_	01-089-8103
	maghemite (Mh)	γ-Fe_2_O_3_	01-089-3850
	quartz (Q)	SiO_2_	01-083-0542
(c)	hematite (H)	α-Fe_2_O_3_	01-089-8103
	maghemite (Mh)	γ-Fe_2_O_3_	01-089-3850
	coesite (C)	SiO_2_	01-076-1805
	anglesite (A)	PbSO_4_	01-072-1389
(d)	fayalite (F)	Fe_2_SiO_4_	01-071-1667
	magnetite (M)	Fe_3_O_4_	01-075-1609
	hematite (H)	α-Fe_2_O_3_	01-089-8103
	cristobalite (C)	SiO_2_	01-076-0936
	franklinite (zinc iron oxide) (Z)	Zn_0.945_Fe_1.78_O_3.71_	01-087-1230
	sillimanite (S)	Al_2_SiO_5_	01-088-0893
(e)	fayalite (F)	Fe_2_SiO_4_	01-071-1667
	magnetite (M)	Fe_3_O_4_	01-075-1609
	hematite (H)	α-Fe_2_O_3_	01-089-8103
	gunningite (Gu)	ZnSO_4_ H_2_O	00-012-0781
	szomolnikite (Sz)	FeSO_4_ H_2_O	00-001-0612
SFT sample	fayalite (F)	Fe_2_SiO_4_	01-071-1667
	magnetite (M)	Fe_3_O_4_	01-075-1609
	cristobalite (C)	SiO_2_	01-076-0936
	franklinite (zinc iron oxide) (Z)	(Zn_0.984_Fe_0.015_)Fe_1.953_O_3.938_	01-087-1230
	illite (clay-mica) (I)	KAl_2_(Si_3_Al)O_10_(OH)_2_	00-043-0685

**Figure 10:**
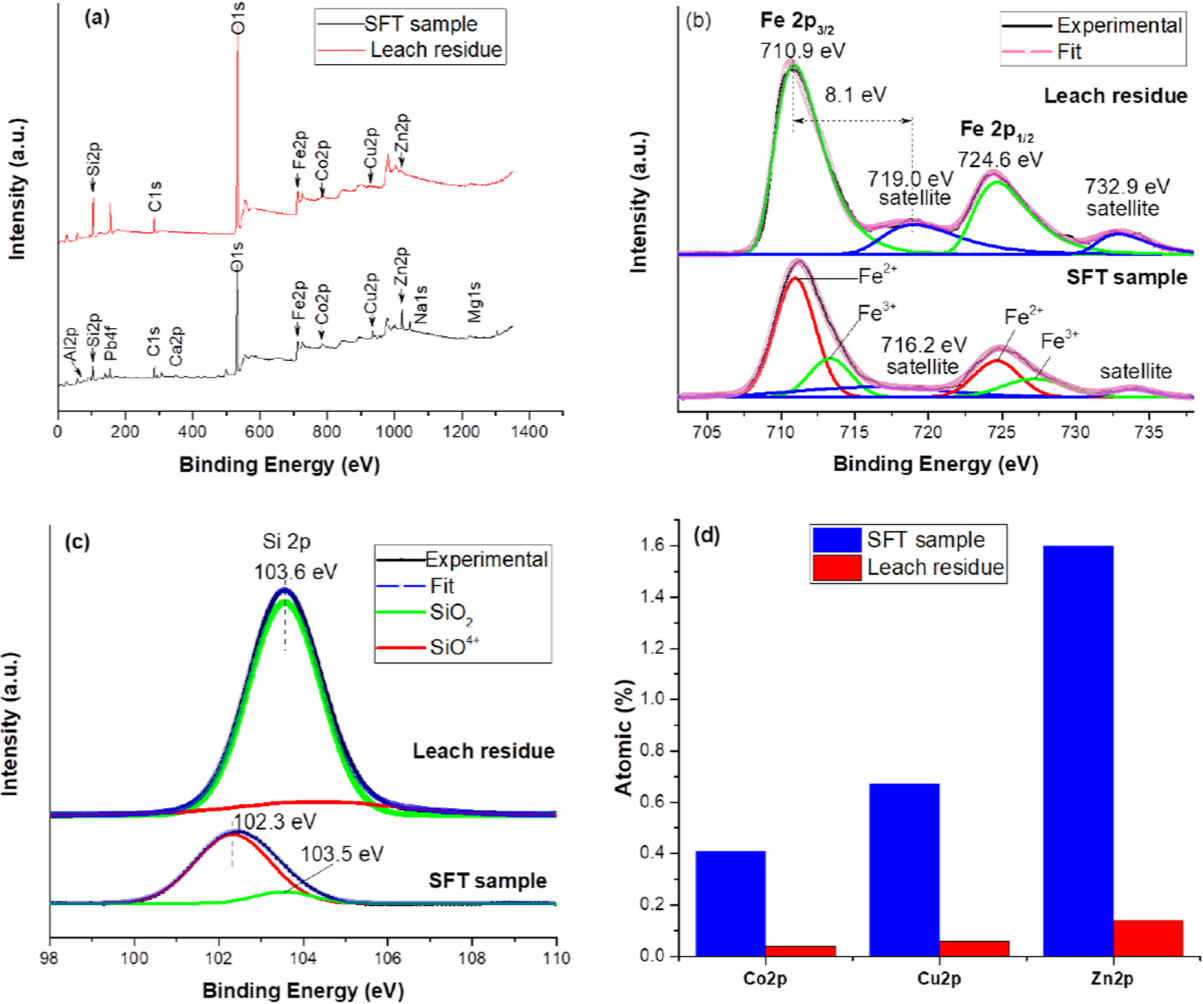
XPS spectra of the SFT sample and the leaching residue obtained under the optimal pressure leaching conditions. (a) Survey scan and high-resolution scans in the (b) Fe 2p and (c) Si 2p regions and (d) atomic percentages of Co 2p, Cu 2p, and Zn 2p on the surface before and after leaching.

The SFT sample and the leaching residue obtained under the optimal leaching conditions were also analyzed using XPS to investigate the chemical changes^77−81^ involved in the oxidative pressure leaching process. The survey XPS spectra of the samples are represented in Figure 10a. To confirm the formation of Fe_2_O_3_ during the leaching of the SFT sample, the high-resolution photoelectron spectrum of Fe 2p was collected and is shown in Figure 10b. The Fe 2p spectrum was fitted, and the results after subtraction of the background are shown. Figure 10b shows the spectra of Fe 2p (SFT sample), where the binding energy (BE) of 710.9 eV is attributed to Fe^2+^ (fayalite), while the peak at 713.3 eV corresponds to Fe^3+^ due to the presence of magnetite and franklinite^82−84^ in the SFT sample. The Fe 2p_3/2_–Fe 2p_1/2_ binding energies of Fe^2+^ ions in fayalite were reported to be 709–722.6,^85^ 710.7–724,^77^ 711.1–724.6,^86^ and 709.7–723^87^ eV. Satellite peaks are generally used to derive information regarding oxidation states. A weak satellite peak in the SFT sample was recorded. However, significant satellite peaks appeared at 719.0 and 732.9 eV in the leaching residue due to the formation of hematite. For the leaching residue (Figure 10b), the main Fe 2p_3/2_ peak and the Fe 2p_1/2_ peak had BEs of 710.9 and 724.6 eV, respectively. These results indicate that iron was completely in the Fe^3+^ state. This finding was also confirmed by the XRD results (see Figure 10b), which indicated that α-Fe_2_O_3_ and a small amount of γ-Fe_2_O_3_ (maghemite) contained only Fe^3+^ cations. It is also worth noting that the BE separation between the satellite peak and Fe 2p_3/2_ in the SFT sample (mainly fayalite) was 5.2 eV, while the BE separation of hematite was 8.1 eV. In other words, the satellite peak of Fe 2p_3/2_ was located approximately 8.1 eV higher than the main Fe 2p_3/2_ peak, agreeing well with the values reported for hematite.^85,87−91^ The BE of the Si 2p peaks for the SFT sample was located at 102.3 eV, which corresponds to silicate (Fe_2_SiO_4_), while the BE of 103.5 eV indicates quartz (SiO_2_), which is consistent with the corresponding XRD diagram (see Figure 3). Figure 10c also shows that the BE of Si 2p shifted to a higher value (103.6 eV) after pressure leaching, indicating that amorphous SiO_2_ was generated in the process. Figure 10d presents the changes in the atomic percentages of Co, Cu, and Zn before and after the leaching process. The atomic percentages for Co 2p, Cu 2p, and Zn 2p decreased from 0.41, 0.67, and 1.6% to 0.04, 0.06, and 0.14%, respectively, indicating a high leaching efficiency.

## 4 Conclusions

The effects of the main parameters, such as sulfuric acid concentration, leaching temperature, oxygen partial pressure, leaching time, and solid/liquid (*S*/*L*) ratio, on the oxidative pressure acid leaching of copper slag flotation tailings (SFT) were determined. Pressure oxidative acid leaching, which has some advantages, such as selective dissolution, high leaching efficiency, and environmental considerations, was successfully applied to the SFT sample. It was possible to recover more than 90% of base metals with a single-step leaching process. In particular, cobalt is a metal that is critical in battery storage for electric vehicles as well as other storage applications. Since cobalt has been deemed a “critical metal”, it is important to effectively evaluate cobalt-bearing tailings.

The results of the leaching investigations showed that oxygen partial pressure, sulfuric acid concentration, and *S*/*L* ratio have significant effects on the extraction of cobalt, copper, and zinc. The presence of oxygen in this leaching system is crucial since it enhances the dissolution of copper, cobalt, zinc, and the oxidation of sulfides as well as the transformation of iron in fayalite, magnetite, or franklinite to hematite. The extraction efficiencies of Co, Cu, Zn, and Fe were only 24.5, 5.3, 26.3, and 13.1%, respectively, without oxygen supply. Additionally, high acid concentrations and high *S*/*L* ratios lead to silica gel formation, which causes filtration problems and inhibits metal dissolution. The considerable decrease in the extraction efficiencies of Co, Cu, and Zn at *S*/*L* ratios of 2:5 and 2.5:5 might be due to the combined effect of a higher slurry viscosity, less dissolved oxygen, and the formation of a larger quantity of gelatinous material, thus coating the particles.

High extraction efficiencies of 96.3 ± 1.8% for cobalt, 93.1 ± 1.1% for copper, and 92.3 ± 1.7% for zinc were achieved under the optimum leaching conditions (H_2_SO_4_ = 250 kg/t, *S*/*L* = 1:5, *P*_O_2__ = 0.7 MPa, τ = 60 min, stirring rate of 500 rpm, *d*_80_ = 58 μm). The extraction efficiency of iron was only 0.49 ± 0.12%. Compared to the results reported in previous studies, the optimized conditions cause high selective leaching of cobalt, copper, and zinc compared to iron, strongly indicating the thorough removal of iron from the leaching liquor. These metals in the leaching solution are easily recovered and separated by traditional processes such as ion exchange and solvent extraction. The final residue obtained under the optimum leaching conditions consisted mainly of ∼56 wt % hematite (α-Fe_2_O_3_) and amorphous SiO_2_, indicating that the leaching residue can be safely stored or further used in the steel and iron or cement industries.

## References

[ref1] U.S. Geological Survey Mineral commodity summaries 2022: U.S. Geological Survey, 2022, p 202 https://pubs.usgs.gov/periodicals/mcs2022/mcs2022.pdf.

[ref2] Gorai B.; Jana R. K.; Premchand Characteristics and utilization of copper slag—a review. Resour. Conserv. Recycl. 2003, 39, 299–313. 10.1016/S0921-3449(02)00171-4.

[ref3] Edraki M.; Baumgartl T.; Manlapig E.; Bradshaw D.; Franks D. M.; Moran C. J. Designing mine tailings for better environmental, social and economic outcomes: A review of alternative approaches. J. Cleaner Prod. 2014, 84, 411–420. 10.1016/j.jclepro.2014.04.079.

[ref4] Wang X.; Geysen D.; van Gerven T.; Blanpain B. Characterization of Copper Slag. In REWAS 2013; Springer: Cham, Switzerland, 2013; pp 54–68.

[ref5] Gonzalez C.; Parra R.; Klenovcanova A.; Imris I.; Sánchez M. Reduction of Chilean copper slags: A case of waste management project. Scand. J. Metallurgy. 2005, 34, 143–149. 10.1111/j.1600-0692.2005.00740.x.

[ref6] Shen H.; Forssberg E. An overview of recovery of metals from slags. Waste Manage. 2003, 23, 933–949. 10.1016/S0956-053X(02)00164-2. 14614927

[ref7] Madheswaran C. K.; Ambily P. S.; Dattatreya J. K.; Rajamane N. P. Studies on use of copper slag as replacement material for river sand in building constructions. J. Inst. Eng. (India): Ser. A 2014, 95, 169–177. 10.1007/s40030-014-0084-9.

[ref8] Gabasiane T. S.; Danha G.; Mamvura T. A.; Mashifana T.; Dzinomwa G. Environmental and socioeconomic impact of copper slag–a review. Crystals 2021, 11, 1504 10.3390/cryst11121504.

[ref9] Mohapatra B. K.; Nayak B. D.; Rao G. V. Microstructure of and metal distribution in indian copper converter slags study bu scanning-electron microscopy. Trans. Instn. Min. Metall. (Sect. C: Mineral Process. Extr. Metall.) 1994, 103, C217–C220.

[ref10] Arslan C.; Arslan F. Recovery of copper, cobalt and zinc from copper smelter and converter slag. Hydrometallurgy 2002, 67, 1–7. 10.1016/S0304-386X(02)00139-1.

[ref11] Kokal H. R. Fluxes for Metallurgy. In Industrial Minerals and Rocks: Commodities, Markets, and Uses, 7th ed.; Kogel J. E.; Trivedi N. C.; Barker J. M.; Krukowski S. T., Eds.; Society for Mining Metallurgy and Exploration, Inc.: Littleton, Colorado, 2006; pp 1405–1422.

[ref12] Syed S. A green technology for recovery of gold from non-metallic secondary sources. Hydrometallurgy 2006, 82, 48–53. 10.1016/j.hydromet.2006.01.004.

[ref13] Havlik T.; Orac D.; Petranikova M.; Miskufova A.; Kukurugya F.; Takacova Z. Leaching of copper and tin from used printed circuit boards after thermal treatment. J. Hazard. Mater. 2010, 183, 866–873. 10.1016/j.jhazmat.2010.07.107. 20800354

[ref14] Akcil A.; Vegliò F.; Ferella F.; Okudan M. D.; Tuncuk A. A review of metal recovery from spent petroleum catalysts and ash. Waste Manage. 2015, 45, 420–433. 10.1016/j.wasman.2015.07.007. 26188611

[ref15] Chen X.; Ma H.; Luo C.; Zhou T. Recovery of valuable metals from waste cathode materials of spent lithium-ion batteries using mild phosphoric acid. J. Hazard. Mater. 2017, 326, 77–86. 10.1016/j.jhazmat.2016.12.021. 27987453

[ref16] Saguru C.; Ndlovu S.; Moropeng D. A review of recent studies into hydrometallurgical methods for recovering PGMs from used catalytic converters. Hydrometallurgy 2018, 182, 44–56. 10.1016/j.hydromet.2018.10.012.

[ref17] Zheng X.; Zhu Z.; Lin X.; Zhang Y.; He Y.; Cao H.; Sun Z. A mini-review on metal recycling from spent lithium ion batteries. Engineering 2018, 4, 361–370. 10.1016/j.eng.2018.05.018.

[ref18] Moosakazemi F.; Mohammadi M.R.T.; Zakeri M.; Esmaeili M. J.; Rafiei H. Development of an environmentally friendly flowsheet for the hydrometallurgical recovery of nickel and aluminum from spent methanation catalyst. J. Cleaner Prod. 2020, 244, 118731 10.1016/j.jclepro.2019.118731.

[ref19] Wiecka Z.; Rzelewska-Piekut M.; Cierpiszewski R.; Staszak K.; Regel-Rosocka M. Hydrometallurgical recovery of cobalt(II) from spent industrial catalysts. Catalysts 2020, 10, 61 10.3390/catal10010061.

[ref20] Binnemans K.; Jones P. T.; Manjón Fernández Á.; Torres V. M. Hydrometallurgical processes for the recovery of metals from steel industry by-products: A critical review. J. Sustainable Metall. 2020, 6, 505–540. 10.1007/s40831-020-00306-2.

[ref21] Larouche F.; Tedjar F.; Amouzegar K.; Houlachi G.; Bouchard P.; Demopoulos G. P.; Zaghib K. Progress and status of hydrometallurgical and direct recycling of Li-ion batteries and beyond. Materials 2020, 13, 801 10.3390/ma13030801. PMC7040742 32050558

[ref22] Le M. N.; Lee M. S. A review on hydrometallurgical processes for the recovery of valuable metals from spent catalysts and life cycle analysis perspective. Miner. Process. Extr. Metall. Rev. 2021, 42, 335–354. 10.1080/08827508.2020.1726914.

[ref23] Lin S.-S.; Chiu K.-H. An evaluation of recycling schemes for waste dry batteries–a simulation approach. J. Cleaner Prod. 2015, 93, 330–338. 10.1016/j.jclepro.2015.01.045.

[ref24] Özcan Ö. F.; Karadağ T.; Altuğ M.; Özgüven Ö. F. [A review study on the characteristics and advantages of battery chemicals used in electric vehicles] Elektrikli araçlarda kullanılan pil kimyasallarının özellikleri ve üstün yönlerinin kıyaslanması üzerine bir derleme çalışması GU J. Sci., Part A, 2021; Vol. 8 (2), , pp 276–298.

[ref25] Anand S.; Rao P. K.; Jena P. K. Recovery of metal values from copper converter and smelter slags by ferric chloride leaching. Hydrometallurgy 1980, 5, 355–365. 10.1016/0304-386X(80)90025-0.

[ref26] Anand S.; Das R. P.; Jena P. K. Reduction-roasting and ferric chloride leaching of copper converter slag for extraction copper, nickel and cobalt values. Hydrometallurgy 1981, 7, 243–252. 10.1016/0304-386X(81)90005-0.

[ref27] Beşe A. V. Effect of ultrasound on the dissolution of copper from copper converter slag by acid leaching. Ultrason. Sonochem. 2007, 14, 790–796. 10.1016/j.ultsonch.2007.01.007. 17383213

[ref28] Carranza F.; Iglesias N.; Mazuelos A.; Romero R.; Forcat O. Ferric leaching of copper slag flotation tailings. Miner. Eng. 2009, 22, 107–110. 10.1016/j.mineng.2008.04.010.

[ref29] Burzynska L.; Gumowska W.; Rudnik E.; Partyka J. Mechanism of the anodic dissolution of Cu70-Co4-Fe14-Pb7 alloy originated from reduced copper converter slag in an ammoniacal solution. Recovery of copper and cobalt. Hydrometallurgy 2008, 92, 34–41. 10.1016/j.hydromet.2008.01.009.

[ref30] Herreros O.; Quiroz R.; Manzano E.; Bou C.; Vinals J. Copper extraction from reverberatory and flash furnace slags by chlorine leaching. Hydrometallurgy 1998, 49, 87–101. 10.1016/S0304-386X(98)00010-3.

[ref31] Beşe A. V.; Ata O. N.; Çelik C.; Çolak S. Determination of the optimum conditions of dissolution of copper in converter slag with chlorine gas in aqueous media. Chem. Eng. Process. 2003, 42, 291–298. 10.1016/S0255-2701(02)00040-5.

[ref32] Anand S.; Rao K. S.; Jena P. K. Pressure leaching of copper converter slag using dilute sulphuric acid for the extraction of cobalt, nickel and copper values. Hydrometallurgy 1983, 10, 305–312. 10.1016/0304-386X(83)90061-0.

[ref33] Basir S. A.; Rabah M. A. Hydrometallurgical recovery of metal values from brass smelting slag. Hydrometallurgy 1999, 53, 31–44. 10.1016/S0304-386X(99)00030-4.

[ref34] Banza A. N.; Gock E.; Kongolo K. Base metals recovery from copper smelter slag by oxidizing leaching and solvent extraction. Hydrometallurgy 2002, 67, 63–69. 10.1016/S0304-386X(02)00138-X.

[ref35] Altundogan H. S.; Boyrazli M.; Tumen F. A study on the sulphuric acid leaching of copper converter slag in the presence of dichromate. Miner. Eng. 2004, 17, 465–467. 10.1016/j.mineng.2003.11.002.

[ref36] Bulut G.; Gül A.; Kangal O.; Önal G. Evaluation of Metallic Values from Ancient Slags. In COM, The Conference of Metallurgists. Materials: The Future of Manufacturing in A Sustainable Environment. Proceedings of the Fifth International Symposium on Waste Processing and Recycling in Mineral and Metallurgical Industries, Ontario, Canada, 2004; pp 22–25.

[ref37] Baghalha M.; Papangelakis V. G.; Curlook W. Factors affecting the leachability of Ni/Co/Cu slags at high temperature. Hydrometallurgy 2007, 85, 42–52. 10.1016/j.hydromet.2006.07.007.

[ref38] Ahmed I. M.; Nayl A. A.; Daoud J. A. Leaching and recovery of zinc and copper from brass slag by sulfuric acid. J. Saudi Chem. Soc. 2016, 20, S280–S285. 10.1016/j.jscs.2012.11.003.

[ref39] Dimitrijevic M. D.; Urosevic D. M.; Milic S. M.; Sokic M. D.; Markovic R. T. Dissolution of copper from smelting slag by leaching in chloride media. J. Min. Metall., Sect. B 2017, 53, 407 10.2298/JMMB170425016D.

[ref40] Tshiongo N.; Mbaya R. K. K.; Maweja K.; Tshabalala L. C. Effect of cooling rate on base metals recovery from copper matte smelting slags. World Academy of Science, Engineering and Technology 2010, 70, 273–277. 10.5281/zenodo.1085311.

[ref41] Tshiongo N.; Mbaya R.K.K.; Maweja K. Leaching Kinetics of Cu, Co, Zn, Pb and Fe from Copper Smelting Slags Cooled in Different Ways After Tapping. In The Southern African Institute of Mining and Metallurgy, 6th Southern African Base Metals Conference, Phalaborwa, South Africa, 2011; pp 463–476.

[ref42] Jafarifar D.; Daryanavard M. R.; Sheibani S. Ultra fast microwaveassisted leaching for recovery of platinum from spent catalyst. Hydrometallurgy 2005, 78, 166–171. 10.1016/j.hydromet.2005.02.006.

[ref43] Sheng P. P.; Etsell T. H. Recovery of gold from computer circuit board scrap using aqua regia. Waste Manage. Res. 2007, 25, 380–383. 10.1177/0734242X07076946. 17874665

[ref44] Willner J.; Fornalczyk A.; Cebulski J.; Janiszewski K. Preliminary studies on simultaneous recovery of precious metals from different waste materials by pyrometallurgical method. Arch. Metall. Mater. 2014, 59, 801–804. 10.2478/amm-2014-0136.

[ref45] Bang J.-H.; Lee S.-W.; Jeon C.; Park S.; Song K.; Jo W. J.; Chae S. Leaching of metal ions from blast furnace slag by using aqua regia for CO_2_ mineralization. Energies 2016, 9, 996 10.3390/en9120996.

[ref46] Halli P.; Wilson B. P.; Hailemariam T.; Latostenmaa P.; Yliniemi K.; Lundström M. Electrochemical recovery of tellurium from metallurgical industrial waste. J. Appl. Electrochem. 2020, 50, 1–14. 10.1007/s10800-019-01363-6.

[ref47] Halli P.; Hailemariam T.; Latostenmaa P.; Lundström M. Leaching behavior of Cu, Bi And Sb from Trof furnace doré slag during mineral acid leaching. In International Mineral Processing Congress (IMPC 2018); Russian Federation: Moscow, 2018; pp 446–454 978-1-5108-7499-2.

[ref48] Nadirov R. K.; Syzdykova L. I.; Zhussupova A. K.; Usserbaev M. T. Recovery of value metals from copper smelter slag by ammonium chloride treatment. Int. J. Miner. Process. 2013, 124, 145–149. 10.1016/j.minpro.2013.07.009.

[ref49] Altundoğan H. S.; Tümen F. Metal recovery from copper converter slag by roasting with ferric sulphate. Hydrometallurgy 1997, 44, 261–267. 10.1016/S0304-386X(96)00038-2.

[ref50] Sukla L. B.; Panda S. C.; Jena P. K. Recovery of cobalt, nickel and copper from converter slag through with ammonium sulphate and sulphuric acid. Hydrometallurgy 1986, 16, 153–165. 10.1016/0304-386X(86)90040-X.

[ref51] Çakır M.; Kartal M.; Gül H.; Taşkın E.; Uysal M.; Aydın A. O.; Alp A. Copper Recovery from Copper Refining Slag Flotation Waste Bakır rafinasyon curufu flotasyon atıklarındaki bakırın geri kazanımı, 1st International Symposium on Innovative Technologies in Engineering and Science, Sakarya University, Sakarya, 7-9 June, 2013; pp 200–207.

[ref52] Ziyadanoğulları B. Recovery of copper and cobalt from concentrate and converter slag. Sep. Sci. Technol. 2000, 35, 1963–1971. 10.1081/SS-100100630.

[ref53] Deng T.; Ling Y. Processing of copper converter slag for metal reclamation. Part I: extraction and recovery of copper and cobalt. Waste Manage. Res. 2007, 25, 440–448. 10.1177/0734242X07077613. 17985669

[ref54] Tümen F.; Bailey N. T. Recovery of metal values from copper smelter slags by roasting with pyrite. Hydrometallurgy 1990, 25, 317–328. 10.1016/0304-386X(90)90047-6.

[ref55] Dimitrijevic M. D.; Urosevic D. M.; Jankovic Z. D.; Milic S. M. Recovery of copper from smelting slag by sulphation roasting and water leaching. Physicochem. Probl. Miner. Process. 2016, 52, 409–421. 10.5277/ppmp160134.

[ref56] Yang Z.; Rui-lin M.; Wang-dong N.; Hui W. Selective leaching of base metals from copper smelter slag. Hydrometallurgy 2010, 103, 25–29. 10.1016/j.hydromet.2010.02.009.

[ref57] Boyrazlı M.; Altundoğan H. S.; Tümen F. Recovery of metals from copper converter slag by leaching with K_2_Cr_2_O_7_-H_2_SO_4_. Can. Metall. Q. 2006, 45, 145–152. 10.1179/cmq.2006.45.2.145.

[ref58] Yiğit Y.; Kuzu M.; Yazıcı E. Y.; Celep O.; Deveci H. Leaching of metals from flotation tailings of a copper smelter slag in acidic solutions. In New Trends in Mining, Proceeding of 25th International Mining Congress of Turkey, IMCET, Bayraktar İ.; Karadeniz M.; Kılıç M. G.; Atalay F., Eds.; 2017; pp 614–622.

[ref59] Nadirov R. K.; Mussapyrova L. A. Copper smelter slag leaching by using H_2_SO_4_ in the presence of dichromate. J. Chem. Technol. Metall. 2019, 54, 657–662.

[ref60] Perederiy I.; Papangelakis V. G.; Buarzaiga M.; Mihaylov I. Co-treatment of converter slag and pyrrhotite tailings via high pressure oxidative leaching. J. Hazard. Mater. 2011, 194, 399–406. 10.1016/j.jhazmat.2011.08.012. 21893384

[ref61] Li Y.; Papangelakis V. G.; Perederiy I. High pressure oxidative acid leaching of nickel smelter slag: Characterization of feed and residue. Hydrometallurgy 2009, 97, 185–193. 10.1016/j.hydromet.2009.03.007.

[ref62] Potysz A.; van Hullebusch E. D.; Kierczak J.; Grybos M.; Lens P. N.; Guibaud G. Copper metallurgical slags – Current knowledge and fate: A review. Crit. Rev. Environ. Sci. Technol. 2015, 45, 2424–2488. 10.1080/10643389.2015.1046769.

[ref63] Tian H.; Guo Z.; Pan J.; Zhu D.; Yang C.; Xue Y.; Li S.; Wang D. Comprehensive review on metallurgical recycling and cleaning of copper slag. Resour., Conserv. Recycl. 2021, 168, 105366 10.1016/j.resconrec.2020.105366.

[ref64] Roy S.; Datta A.; Rehani S. Flotation of copper sulphide from copper smelter slag using multiple collectors and their mixtures. Int. J. Miner. Process. 2015, 143, 43–49. 10.1016/j.minpro.2015.08.008.

[ref65] Bulut G.; Perek K. T.; Gül A.; Arslan F.; Önal G. Recovery of metal values from copper slags by flotation and roasting with pyrite. Min., Metall., Explor. 2007, 24, 13–18. 10.1007/BF03403353.

[ref66] Bruckard W. J.; Somerville M.; Hao F. The recovery of copper, by flotation, from calcium-ferrite-based slags made in continuous pilot plant smelting trials. Miner. Eng. 2004, 17, 495–504. 10.1016/j.mineng.2003.12.004.

[ref67] Osborn G. A.; Garner F. A.; Veasey T. J. Recovery of Metal Values from Secondary Copper Slags, In Proceedings of the 1st International Mineral Processing Symposium, Izmir, Turkey, 29 September-1 October, 1986; pp 46–64.

[ref68] Sarrafi A.; Rahmati B.; Hassani H. R.; Shirazi H. H. Recovery of copper from reverberatory furnace slag by flotation. Miner. Eng. 2004, 17, 457–459. 10.1016/j.mineng.2003.10.018.

[ref69] Arslan F.; Giray K.; Önal G.; Gürkan V. Development of a flowsheet for recovering copper and tin from copper refining slags. Eur. J. Miner. Process. Environ. Prot. 2002, 2, 94–102.

[ref70] Muravyov M. I.; Fomchenko N. V.; Usoltsev A. V.; Vasilyev E. A.; Kondrat’eva T. F. Leaching of copper and zinc from copper converter slag flotation tailings using H_2_SO_4_ and biologically generated Fe_2_(SO_4_)_3_. Hydrometallurgy 2012, 119–120, 40–46. 10.1016/j.hydromet.2012.03.001.

[ref71] Urosevic D. M.; Dimitrijevic M. D.; Jankovic Z. D.; Antic D. V. Recovery of copper from copper slag and copper slag flotation tailings by oxidative leaching. Physicochem. Probl. Miner. Process. 2015, 51, 73–82. 10.5277/ppmp150107.

[ref72] Potysz A.; Kierczak J. Prospective (bio)leaching of historical copper slags as an alternative to their disposal. Minerals 2019, 9, 542 10.3390/min9090542.

[ref73] Liao Y.; Ji G.; Shi G.; Xi J. A Study on the selective leaching of valuable metals and the configuration of iron silicon phases in copper smelting slag by oxidative Pressure leaching. J. Sustainable Metallurgy 2021, 7, 1143–1153. 10.1007/s40831-021-00404-9.

[ref74] Li Y.; Perederiy I.; Papangelakis V. G. Cleaning of waste smelter slags and recovery of valuable metals by pressure oxidative leaching. J. Hazard. Mater. 2008, 152, 607–615. 10.1016/j.jhazmat.2007.07.052. 17728060

[ref75] Seyrankaya A.; Canbazoğlu M. Recovery of cobalt, copper and zinc from Küre-Kastamonu historical copper slag by high pressure oxidative acid leaching. Russ. J. Non-Ferrous. Met. 2021, 62, 390–402. 10.3103/S1067821221040131.

[ref76] Shi G.; Liao Y.; Su B.; Zhang Y.; Wang W.; Xi J. Kinetics of copper extraction from copper smelting slag by pressure oxidative leaching with sulfuric acid. Sep. Purif. Technol. 2020, 241, 116699 10.1016/j.seppur.2020.116699.

[ref77] Chowdhury S. R.; Yanful E. K.; Pratt A. R. Recycling of nickel smelter slag for arsenic remediation—an experimental study. Environ. Sci. Pollut. Res. 2014, 21, 10096–10107. 10.1007/s11356-014-2892-x. 24770924

[ref78] Guo P.; Wang C. Good lithium storage performance of Fe_2_SiO_4_ as an anode material for secondary lithium ion batteries. RSC Adv. 2017, 7, 4437–4443. 10.1039/C6RA26376C.

[ref79] Xu D.-M.; Fu R.-B. The mechanistic understanding of potential bioaccessibility of toxic heavy metals in the indigenous zinc smelting slags with multidisciplinary characterization. J. Hazard. Mater. 2022, 425, 127864 10.1016/j.jhazmat.2021.127864. 34915297

[ref80] Wang Z.; Zhao Z.; Zhang L.; Liu F.; Peng B.; Chai L.; Liu D.; Liu D.; Wang T.; Liu H.; Liang Y. Formation mechanism of zinc-doped fayalite (Fe_2-x_Zn_x_SiO_4_) slag during copper smelting. J. Hazard. Mater. 2019, 364, 488–498. 10.1016/j.jhazmat.2018.10.071. 30388632

[ref81] Tao L.; Wang L.; Yang K.; Wang X.; Chen L.; Ning P. Leaching of iron from copper tailings by sulfuric acid: behavior, kinetics and mechanism. RSC Adv. 2021, 11, 5741–5752. 10.1039/D0RA08865J. 35423117 PMC8694737

[ref82] Li M.; Peng B.; Chai L.; Peng N.; Xie X.; Yan H. Technological mineralogy and environmental activity of zinc leaching residue from zinc hydrometallurgical process. Trans. Nonferrous Met. Soc. China 2013, 23, 1480–1488. 10.1016/S1003-6326(13)62620-5.

[ref83] Yao J.; Yan J.; Huang Y.; Li Y.; Xiao S.; Xiao J. Preparation of ZnFe_2_O_4_/α-Fe_2_O_3_ nanocomposites from sulfuric acid leaching liquor of jarosite residue and their application in lithium-ion batteries. Front. Chem. 2018, 6, 442 10.3389/fchem.2018.00442. 30320073 PMC6167419

[ref84] Fan G.; Gu Z.; Yang L.; Li F. Nanocrystalline zinc ferrite photocatalysts formed using the colloid mill and hydrothermal technique. Chem. Eng. J. 2009, 155, 534–541. 10.1016/j.cej.2009.08.008.

[ref85] Yamashita T.; Hayes P. Analysis of XPS spectra of Fe^2+^ and Fe^3+^ ions in oxide materials. Appl. Surf. Sci. 2008, 254, 2441–2449. 10.1016/j.apsusc.2007.09.063.

[ref86] Wang D.; Peng N.; Zhao Z.; Peng B.; Wang Z.; Gong D. Investigation into the function of zero-valent iron (ZVI) in the process of fayalite formation. JOM 2020, 72, 2721–2729. 10.1007/s11837-020-04182-9.

[ref87] Hirokawa K.; Oku M. Application of ESCA to semi-quantitative surface and state analysis of iron oxides. Talanta 1979, 26, 855–859. 10.1016/0039-9140(79)80265-9. 18962526

[ref88] Mills P.; Sullivan J. L. A study of the core level electrons in iron and its three oxides by means of X-ray photoelectron spectroscopy. J. Phys. D: Appl. Phys. 1983, 16, 723 10.1088/0022-3727/16/5/005.

[ref89] Hawn D. D.; DeKoven B. M. Deconvolution as a correction for photoelectron inelastic energy losses in the core level XPS spectra of iron oxides. Surf. Interface Anal. 1987, 10, 63–74. 10.1002/sia.740100203.

[ref90] Muhler M.; Schlögl R.; Ertl G. The nature of the iron oxide-based catalyst for dehydrogenation of ethylbenzene to styrene 2. Surface chemistry of the active phase. J. Catal. 1992, 138, 413–444. 10.1016/0021-9517(92)90295-S.

[ref91] Grosvenor A. P.; P Kobe B. A.; Biesinger M. C.; McIntyre N. S. Investigation of multiplet splitting of Fe 2p XPS spectra and bonding in iron compounds. Surf. Interface Anal. 2004, 36, 1564–1574. 10.1002/sia.1984.

